# Hyporeninemic hypoaldosteronism in *RMND1*-related mitochondrial disease

**DOI:** 10.1007/s00467-023-06079-6

**Published:** 2023-07-14

**Authors:** Martin Kömhoff, Valentina Gracchi, Henry Dijkman, Bodo B. Beck, Leo Monnens

**Affiliations:** 1 University Children’s Hospital, Philipps University, Marburg, Germany; 2 Department of Pediatrics, UMCG, University Groningen, Groningen, the Netherlands; 3 Department of Pathology, Radboud University Centre, Nijmegen, the Netherlands; 4 Department of Human Genetics, Cologne, Germany; 5 Department of Physiology, Radboud University Centre, Nijmegen, the Netherlands

**Keywords:** *RMND1* mutations, Hyporeninemic hypoaldosteronism, Inherited renal disease, Mitochondrial dysfunction

## Abstract

**Background:**

*RMND1* is a nuclear gene needed for proper function of mitochondria. A pathogenic gene will cause multiple oxidative phosphorylation defects. A renal phenotype consisting of hyponatremia, hyperkalemia, and acidosis is frequently reported, previously considered to be due to aldosterone insensitivity.

**Methods:**

Clinical features and pathophysiology of three patients will be reported. DNA of these patients was subjected to exome screening.

**Results:**

In the first family, one pathogenic heterozygous and one highly probable heterozygous mutation were detected. In the second family, a homozygous pathogenic mutation was present. The electrolyte disbalance was not due to aldosterone insensitivity but to low plasma aldosterone concentration, a consequence of low plasma renin activity. This disbalance can be treated. In all three patients, the kidney function declined. In the first family, both children suffered from an unexplained arterial thrombosis with dire consequences.

**Conclusions:**

Hyporeninemic hypoaldosteronism is the mechanism causing the electrolyte disbalance reported in patients with *RMND1* mutations, and can be treated.

## Introduction

*RMND1* is a nuclear gene needed for proper function of mitochondria. Its gene product forms a high-molecular-weight complex necessary for mitochondrial translation, possibly by coordinating the assembly or maintenance of the mitochondrial ribosomes. A homozygous pathogenic gene will cause multiple oxidative phosphorylation deficiencies [1–3].

Kidney disease in *RMND1* deficiency and in particular the combination of hyponatremia, hyperkalemia, and metabolic acidosis was extensively reported by Shayota et al. [4]. In their opinion, pseudohypoaldosteronism (aldosterone insensitivity) was the cause of the electrolyte disturbances. In the present study on three affected children, hyporeninemic hypoaldosteronism was found to be the cause of the electrolyte imbalance, showing in addition an intact aldosterone sensitivity.

## Patients and methods

Clinical features and pathophysiology of three patients will be reported. The principles outlined in the Helsinki declaration are carefully followed. Informed consent was provided by the parents.

## Results

### Patient 1

This girl has previously been described [5]. Further follow-up will be described here. Clinical investigation at the age of 7 months revealed spastic quadriplegia, hearing loss, bilateral convergent strabism, and psychomotor retardation. Plasma renin activity and plasma aldosterone were extremely low (Table 1). Serum electrolytes and effect of mineralocorticoid treatment are presented in Table 2. Serum magnesium was normal. Her kidney function gradually declined. Antihypertensive treatment became necessary. Ultrasonography at the age of 8 years revealed small kidneys with two cysts visible in the medulla of the right kidney. In that year, CAPD treatment was started. Neurologically, she remained stable. She was unable to walk; she could understand simple sentences but was unable to speak. Nevertheless, she had a good quality of life. A brain-stem auditory evoked potential showed a delayed response at the right side, without response at the left side. At the age of 10 years, she received a deceased donor kidney transplant. The transplant had to be removed because of the thrombosis of the renal artery 1 month later, although initially the renal perfusion had been normal. This unusual complication and the medical history of the older sister (see below) incited a more extensive investigation of the coagulation system. The bleeding time, the prothrombin time, the activated partial thromboplastin time, the thrombin time, and fibrinogen level were all normal. Antiphospholipid antibodies were negative. No mutation was found in factor V and factor II. Antithrombin III and protein C and S antigen and activity were normal. At 15 years old, while on CAPD, she was admitted because of pneumonia and dehydration. One day later, the right foot became white and pulseless. Heparin was started. Angiography demonstrated a thrombosis of the arteria iliaca externa dextra. Urokinase therapy and later surgical intervention were unsuccessful and the right leg had to be amputated. After recurrent periods of septicemia, she died in the arms of her parents.

**Table 1:** Renin–angiotensin–aldosterone system

Patient 1
Plasma renin activity: 0.9–1.2 ng angiotensin I/ml/h (normal 8.2 ± 3.0) Plasma aldosterone: 7–12 ng/100 ml (normal 95 ± 28) Adapted to age
Patient 3

Plasma renin activity: < 0.1 nmol (normal 3–18 nmol/l/h)

Plasma aldosterone: < 100 pmol/l (normal 833–5830 pmol/l)

Adapted to age

After ACTH administration: 2700 pmol/l

**Table 2:** Serum electrolyte concentrations mEq/l

	Na^+^	K^+^	Cl^−^	Bicarbonate
Patient 1				
No treatment	125–129	5.9–6.3	98–100	14.5–19
Extra NaCl	139	6.6	112	15.3
Extra NaCl and fludrocortisone	142	4.4	100	24.2
Patient 2				
No treatment	135–137	5.2	105	19
Patient 3				
No treatment	128	6.9	97	17
Extra sodium bicarbonate and fludrocortisone	133	3.9	-	-

A limited autopsy restricted to the kidneys was possible. Both kidneys were small and showed by light microscopy in the cortex all the characteristics of a diffuse chronic tubulointerstitial nephritis. A diffuse interstitial fibrosis was present accompanied by a mononuclear cell infiltrate and severe atrophy of the tubules. Several glomeruli were sclerosed, while other glomeruli showed periglomerular sclerosis. In addition, many cysts of variable size were present in the medulla, especially near the corticomedullary border. Prominent medial hypertrophy of intrarenal arterioles was not present. By immunofluorescence, small mesangial deposits of IgM and C3 were seen in some glomeruli. Electron microscopy revealed numerous mitochondria with abnormal shape and inclusions and vacuoles, pronounced in podocytes but also present in distal tubules. During the autopsy procedure, the small size of the ovaries was noticed.

### Patient 2

The older sister of patient 1 had been investigated elsewhere for her mental retardation and neurologic symptoms. No explanation was offered. The healthy parents had two children. At the age of 7, she was seen for the first time at our outpatient clinic. She clearly resembled her younger sister. Spastic quadriplegia, psychomotor retardation, bilateral strabismus convergens, and hearing loss were present. Plasma renin activity was extremely low. Serum electrolyte values are shown in Table 2. Her kidney function was already abnormal with serum creatinine 106 µmol/l. Ultrasonography showed small echogenic kidneys. Gradually, the kidney function declined. At the age of 14 years, CAPD treatment was started. Six months later, she was admitted because of vomiting and dehydration. Although she was adequately rehydrated, she became comatose. CT scan showed hydrocephalus and structural abnormalities in the cerebellum, predicting a poor prognosis. In agreement with the parents, further treatment was stopped and she died.

Autopsy was limited to the brain. The brain showed no signs of swelling. There was a few days old infarction of the right anterolateral medulla oblongata and more recent infarction of the right cerebellar hemisphere due to a recent thrombosis of the right vertebral artery. Recent small hemorrhagic infarctions were seen bilateral in the frontal and frontoparietal and parasagittal cortex.

### Patient 3

The male patient is the third child of healthy non-consanguineous parents born after an uncomplicated pregnancy. He presented at 6 weeks of age with failure to thrive and vomiting. Serum electrolytes at admission are presented in Table 2, showing decreased sodium and bicarbonate and increased potassium levels. Plasma renin activity and aldosterone were extremely low (Table 1). With a combination of sodium bicarbonate and fludrocortisone, normokalemia was obtained. He is mentally retarded with lower limb spasticity, bilateral sensorineural hearing loss, and severely decreased kidney function (CKD4, eGFR 18 ml/min) at 24 years of age, which led to a living-donor transplantation after a short period on hemodialysis at 25 years of life.

Initially, in all 3 patients, urinary microscopy was normal and proteinuria and glucosuria absent.

### Exome sequencing results and interpretation

#### Exome sequencing results and interpretation concerning patients 1 and 2

DNA of the counselee and her parents was subjected to exome sequencing. This “trio-sequencing” approach enables the detection of variants that have occurred de novo in the counselee.

In these data, two variants were detected in one of the genes described in Mendelian-inherited disorders (gene panel version DG-3.2). Additionally, analysis of the exomes sequencing data beyond this gene panel was performed. With the current knowledge, this did not result in the identification of a variant with a likely role in the counselee’s disorder.

c.713A > G p(Asn238Ser) is a pathogenic mutation, heterozygous paternal.

c.571C > G p(Leu191 Val) is a heterozygous maternal. This variant is not present in control populations (Genome Aggregation Database, GnomAD, http://gnomad.broadinstitute.org).

Applying ClinPred with a sensitivity of 0.925 for missense mutations, a strong argument for pathogenicity can be predicted (ACGM classification 4). The potential pathogenicity is 94%. Exome sequencing was performed with Illumina NovaSeq 6000 after exome enrichment.

#### Exome sequencing results and interpretation concerning patient 3

Homozygous c.713 A > G p(Asn238 Ser) is a pathogenic mutation [1, 2].

Exome sequencing was performed on an Illumina HiSeqw-4000 sequencer.

## Discussion

The key finding in our study is the identification of *RMND1* deficiency as a possible genetic cause of hyporeninemic hypoaldosteronism in infancy. The evidence of *RMND1* deficiency results from the genetic findings in patient 3 and is supported by the genetic findings in patients 1 and 2 with both a pathogenic and a highly probable pathogenic mutation. Functional testing of the probable pathogenic mutation according to the technique applied by Janer [1] was not possible due to the lack of muscle tissue or fibroblasts.

Genetic defects associated with hyporeninemic hypoaldosteronism in childhood are rare. Mutations in genes in the renin-angiotensin system are associated with autosomal recessive renal dysgenesis, clinically characterized by persistent anuria and perinatal death. Rarely, these mutations can also be present as a progressive chronic disease [6]. Autosomal dominant tubulointerstitial kidney disease due to *REN* mutation can be present in childhood [7]. Arterial thrombosis observed in patients 1 and 2 has not been described in *RMND1* mutations. Mutations in inherited thrombophilia genes [8, 9] factor V Leiden, prothrombin mutation, protein C deficiency, protein S deficiency, and antithrombin deficiency, however, were absent. Probably, a second hereditary defect was present, which we were unable to detect. Small ovaries observed in patient 1 can reflect a causative role of *RNMD1* mutation in Perrault syndrome [10].

Shayota et al. [4] commented about the clinical presentation of kidney disease including electrolyte imbalance suggesting pseudohypoaldosteronism type 1 reported in 4 patients. Ng et al. [3] collected clinical and laboratory data from 34 patients with recessive mutations in *RMND1*. Persistent hyponatremia and hyperkalemia were reported in 13 of them. In 17 patients, different stages of chronic kidney disease were present, probably using oral K + binders as in our patients in a later phase of their disease. Initial electrolyte imbalance would remain undetected. In our patients, electrolyte abnormalities were caused by a lack of sufficient aldosterone due to low renin activity. Angiotensin II infusion in patient 1 augmented plasma aldosterone concentration [5].

Low plasma aldosterone may explain the observed electrolyte disturbances. Fine tuning of renal Na^+^ and K^+^ excretion occurs in the distal part of the nephron [11, 12]. The aldosterone-sensitive distal nephron (ASDN) comprises the late distal convoluted tubule, connecting tubule, and entire collecting duct. Na^+^ transport in ASDN is mediated by epithelial Na^+^ (ENac). By upregulating ENac, aldosterone induces a lumen-negative transepithelial potential difference favoring K^+^ secretion. In addition, activation of Na^+^-K^+^-ATPase promotes K^+^ entry via the basolateral site into ASDN cells. Beside these effects, aldosterone directly stimulates K^+^ channels apical ROMK and apical BK channels in type α intercalated cells. Aldosterone facilitates H^+^ secretion via H^+^-ATPase in the apical membrane of the α-intercalated cells.

A lack of stimulation by aldosterone will induce hyponatremia by urinary Na^+^ loss, plasma hyperkalemia, and acidosis, as was observed in our patients.

Mitochondrial disorders constitute a group of often misdiagnosed diseases with frequent multisystemic involvement especially the nervous system and muscles. Mitochondria are recognized as key players in genetic kidney disease [13–15]. Renal tubular cells have very high metabolic rates and are rich in mitochondria. Proximal tubulopathy is the most common phenotype reported in mitochondrial disorders as mitochondrial aerobic respiration is required for the energy-consuming task of reabsorption of the majority of glomerular filtrate. Tubulointerstitial nephritis is rarely reported. In our patient and in all reported biopsies of *RMND1*-affected patients, this tubulointerstitial damage is present. Electron microscopy in the patient reported by Gupta et al. [16] showed mildly enlarged mitochondria with a fluffy granular matrix in tubular cells. Niaudet and Rötig [17] reported that in their patients with tubulointerstitial nephritis due to mitochondrial defects, proximal tubular defects were absent. In our patient with pronounced symptoms of hyporeninemic hypoaldosteronism at the age of 7–12 months, a normal function of the proximal tubule and a still normal concentrating capacity were present.

Release of renin from juxtamedullary cells is precisely controlled by several well-characterized factors [18, 19]. The major physiological pathway of this release is the macula densa mechanism. The factor(s) involved in the defective release in *RMND1* deficiency are not known. In patient 1, not only a lower plasma renin concentration but also a decrease of plasma prorenin was measured. Prorenin is produced by principal cells of the collecting duct and secreted into the tubular lumen and constitutively from the kidney [20, 21]. It was suggested (not proven) that prorenin via active action of the prorenin receptor on the basolateral membrane of the macula densa will stimulate the [pro]renin release [22]. Tubular sensing by the macula densa involves apical NaCl transport mechanism mainly via Na–K-2Cl cotransporter. The Na + gradient generated by basolateral Na + /K + -ATPase drives the apical transport. Adequate mitochondrial function via ATP hydrolysis supports Na + /K + -ATPase activity. When mitochondrial function is impaired in *RMND1* at this side, it will only result in an increase of renin release (see the effect of furosemide). The possible effects on renin release by the juxtaglomerular cell are numerous as is show by Schnermann and Briggs [23].

Mitochondrial shape and function are cell-type specific. They are achieved by modulating the dynamic properties of mitochondria including fusion, fission [24] positional tethering, and local metabolic state [25, 26]. They will determine the selective affection of the renin-aldosterone system.

To summarize, we reveal that hyporeninemic hypoaldosteronism is a specific feature of *RNMD1* deficiency allowing adequate treatment of the electrolyte disturbances. Cure of the mitochondrial defect or mitigating its deleterious effect on the interstitium will be the ultimate goal.

## Data Availability

Data is available upon request**.**
